# Ozone fumigation increases the abundance of nutrients in *Brassica* vegetables: broccoli (*Brassica oleracea* var. *italica)* and Chinese cabbage (*Brassica pekinensis*)

**DOI:** 10.1007/s00217-014-2372-z

**Published:** 2014-11-20

**Authors:** Piotr Rozpądek, Michał Nosek, Irenusz Ślesak, Edward Kunicki, Michał Dziurka, Zbigniew Miszalski

**Affiliations:** 1 Institute of Plant Physiology, Polish Academy of Sciences, ul. Niezapominajek 21, 30-239 Krakow, Poland; 2 Institute of Biology, Pedagogical University, ul. Podchorążych 2, 30-084 Kraków, Poland; 3 Department of Vegetable and Medicinal Plants, University of Agriculture, al. 29 Listopada 54, 31-425 Krakow, Poland; 4 Institute of Environmental Sciences, Jagiellonian University, Gronostajowa 7, 30-387 Kraków, Poland; 5 Małopolska Centre of Biotechnology, Jagiellonian University, Gronostajowa 7, 30-387 Kraków, Poland

**Keywords:** Antioxidants, Anthocyanin, Vitamin E, β-Carotene

## Abstract

*B* *rassicaceae* vegetables, among them broccoli and Chinese cabbage, are well recognized due to the nutritional properties. Four-week-old Chinese cabbage and broccoli seedlings were fumigated with O_3_ for 3 days before being transplanted into the field. The effect of O_3_ treatment was determined after reaching marketable quality (ca. 10 weeks). The inflorescences of O_3_-treated broccoli were enriched in vitamin E (α-tocopherol and γ-tocopherol), whereas Chinese cabbage heads had an increased content of anthocyanins and β-carotene. Ozone treatment did not significantly affect the productivity of both examined vegetables.

## Introduction

Out of the numerous species and strains of the *Brassicaceae* family cultivated, broccoli (*Brassica oleracea* var. *italica)* and Chinese cabbage (*Brassica pekinensis*) are emerging as the most willingly consumed in Europe and North America. The rising interest of dietitians, food industry and consumers is drawn by their unique dietary properties, relatively low growth demands and low price. Both vegetables are abundant in protein, minerals (calcium, phosphorus, iron), sulfur-containing compounds [1] and vitamins, such as provitamin A (β-carotene), vitamin C (ascorbate) and vitamin E (tocopherol), with antioxidant properties [2]. The role of antioxidants in plant and animal physiology seems undisputable. In many cases, their abundance determines the plant’s capacity to cope with unfavorable environmental conditions, whereas in humans, they are associated with chronic disease risk reduction [3, 4] including several types of cancer [5], cardio- and cerebro-vascular, ocular and many neurological diseases [2]. Increasing the abundance of antioxidants and other dietary compounds in plants from the *Brassicaceae* family seems to be of significant importance.

Ozone (O_3_) is a model abiotic elicitor of reactive oxygen species (ROS) in plant cells. It enters the leaves through open stomata and due to its high reactivity immediately reacts with components of the apoplastic space generating various ROS and activating detoxification, including enzymatic and non-enzymatic antioxidants and other defense mechanisms [6, 7]. Moderate doses of O_3_ may enhance plant resistance, and thus, utilizing O_3_ in improving the dietary quality of vegetables seems a promising perspective [8]. In the past, O_3_ fumigation has been reported to have a positive impact on plant growth and attempts have been made to utilize O_3_ in plant productivity improvement [9]. Most recently, our studies on white cabbage confirmed the possibility to improve marketable yield by fumigating seedlings with mild doses of O_3_ [10].

## Materials and methods

### Plant material and ozone treatment

*Brassica oleracea* var. *italica* cultivar Monotop F1 and *Brassica pekinensis* cultivar Mirako F1 seeds were sown in a greenhouse to multipots filled with peat substrate. After 4 weeks, seedlings were transferred to closed top plexiglass chambers for ozone fumigation. Ozone (mixed with ambient air) was supplied by the Fischer type 500 M ozone generator (Germany). A constant concentration of 70 µg m^−3^ of O_3_ was controlled by the 49C UV photometric O_3_ analyzer (Thermo Environmental Instruments Inc. USA). Plants were cultivated under a 12-h photoperiod at 400 µmol m^−2^ s^−1^, with a constant day/night temperature of 17/13 °C. The humidity inside fumigation chambers was ca. 60 %. After fumigation, plant seedlings were transferred into the field in Mydlniki, Krakow (50°5′5″N 19°51′8″E). After 10 weeks in agriculture, plants were harvested for analysis. For one sample, two outermost leaves were harvested from three Chinese cabbage plants. For broccoli, three inflorescences were pooled together for one sample. All analyses were performed in three independent experiments.

### Tocopherol and β-carotene content determination

The abundance of tocopherols and β-carotene was measured with HPLC method according to the procedure described previously by Heudi et al. [11] with modifications according to Nosek et al. [12].

### Anthocyanin content determination

Anthocyanin content was determined spectrophotometrically according to the method described by Schmidt and Mohr [13]. Anthocyanins were extracted from 18 % (v/v) 2-propanol containing 1 % (v/v) HCl, and its abundance was determined as the difference between A650 and A535 per gram of fresh weight.

### Statistical analysis

Statistical analysis was performed by Statistica (Statsoft, USA) statistical software. One-way ANOVA followed by Tukey’s HSD multiple range test was used to determine the individual treatment effects at using a significance level of 0.05.

## Results and discussion

**Fig. 1:**
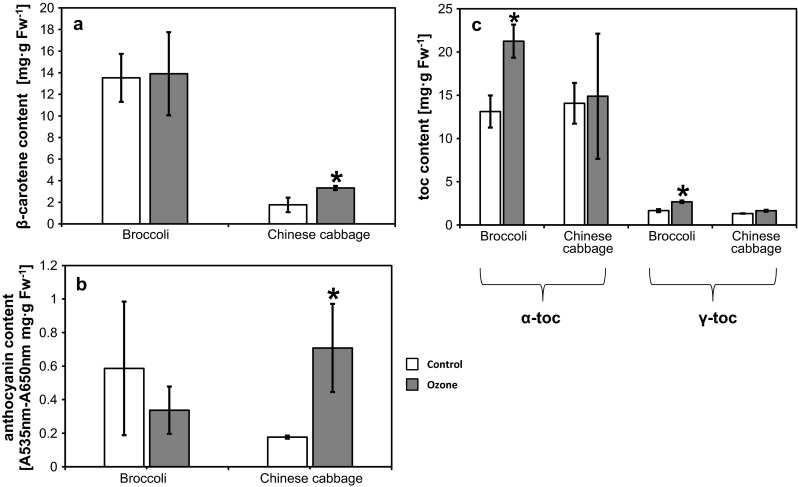
β-carotene (**a**), anthocyanin (**b**) and -tocopherol α and γ (**c**) contents in *Brassica oleracea* var. *italica* and *Brassica pekinensis* treated with 70 µg m^−3^ of O_3_. *Bars* represent mean ± SD of three independent series of measurements of (*white*) control plants and (*gray*) fumigated plants. *Stars above bars* indicate statistically significant differences

Ozone is perceived as a dangerous gaseous pollutant responsible for plant growth limitation and yields loss [9]. Nevertheless, many reports indicate a positive role of episodes of near ambient concentrations of O_3_ on plant growth. As previously reported, doses of up to 150 µg m^−3^ had been utilized to enhance the productivity of plants such as *Phaseolus vulgaris*, natural grassland species, trees and white cabbage [9, 10, 14, 15]. According to the results presented in this communication, episodes of elevated O_3_ during vegetation applied prior to transplantation affect the content of important dietary substances in Chinese cabbage and broccoli. O_3_ treatment had no significant impact on plant productivity as size or marketable yield was unchanged in fumigated plants (data not shown). *B. oleracea* var. *italica* and *B. pekinensis* differ in their long-term response to O_3_ treatment. In inflorescence of broccoli, the abundance of lipophilic, low molecular antioxidants: α-tocopherol and γ-tocopherol, was significantly increased 10 weeks after fumigation (Fig. 1c), but no changes in the content of β-carotene (Fig. 1a) and anthocyanins (Fig. 1b) were observed in O_3_-treated plants. As reported previously, the content of the tocopherols, β-carotene and anthocyanins varies among different cultivars of the same vegetable species. Cultivation conditions, e.g., light quality and quantity are a major factor determining the quantity and composition of important phytochemicals. According to previous reports, the concentration of α- and γ-tocopherol ranges from 22–429 to 2–6, 4 mg g Fw^−1^ for the latter [16, 17]. In the present report, the concentration of α-tocopherol was lower (13 mg g Fw^−1^) compared with literature data; however, O_3_ fumigation significantly increased its content (21, 25 mg g Fw^−1^). The content of γ-tocopherol was in the middle range of the reported concentrations (2–64 mg g Fw^−1^, Kurlich et al. [16]). O_3_ fumigation had a similar impact on its concentration, allowing us to speculate that the abundance of this phytochemical is prone to manipulation. The concentration of other examined substances was in accordance with previously published results [2, 17].

On the contrary, in *B. pekinensis* heads, the content of β-carotene (Fig. 1a) and anthocyanins (Fig. 1b) was increased after fumigation. The relative abundance of anthocyanins was improved over threefold compared with control plants. The concentration of β-carotene after fumigation reached levels (10–40 mg g Fw^−1^) previously reported by Singh et al. [17]. In control plants, its concentration was in the middle range values (17, 6 mg g Fw^−1^) compared with previous reports. Fumigation had no effect on the concentration of tocopherol (Fig. 1c), and it was in accordance with previously reported results [17, 18].

## Conclusions

Treatment of *B. pekinensis* with moderate doses of O_3_ may be utilized in enhancing abundance of both β-carotene and anthocyanins. On the other hand, similar treatment may induce accumulation of α- and γ-tocopherols in *B. oleracea* var. *italic*.
